# Development of a filter device for the prevention of aquatic bacterial disease using a single-chain variable fragment (scFv)-conjugated affinity silk

**DOI:** 10.1038/s41598-022-13408-6

**Published:** 2022-06-08

**Authors:** Harsha Prakash, Mitsuru Sato, Katsura Kojima, Atsushi Sato, Shinpei Maruyama, Takahiro Nagasawa, Miki Nakao, Tomonori Somamoto

**Affiliations:** 1 Laboratory of Marine Biochemistry, Department of Bioscience and Biotechnology, Graduate School of Bioresource and Bioenvironmental Sciences, Kyushu University, Nishi-ku Motooka 744, Fukuoka, 819-0395 Japan; 2 Silkworm Research Group, Division of Silk-Producing Insect Biotechnology, Institute of Agrobiological Sciences, National Agriculture and Food Research Organization, 1-2 Owashi, Tsukuba, Ibaraki 305-8634 Japan; 3 Silk Materials Research Group, Division of Silk-Producing Insect Biotechnology, Institute of Agrobiological Sciences, National Agriculture and Food Research Organization, 1-2 Owashi, Tsukuba, Ibaraki 305-8634 Japan; 4 Kyorin Co. Ltd., 9 Shirogane-machi, Himeji, Hyogo 670-0902 Japan

**Keywords:** Immunological techniques, Antibody generation, ELISA, Animal biotechnology, Biomaterials, Biomaterials - proteins, Pathogens

## Abstract

Infectious disease is one of the most serious problems in the aquaculture industry for ornamental or edible fish. This study attempted to develop a new device for preventing an aquatic bacterial disease, ulcer disease, caused by *Aeromonas salmonicida* (*As*), using “affinity silk”. Affinity silk is a silk protein-containing fibroin L-chain (FibL) fused to the single-chain variable fragment (scFv). It can be easily processed into different formats such as fibers, gels, sponges, or films. A transgenic silkworm that could express a cDNA construct containing FibL fused to an scFv derived from a monoclonal antibody (MAb) against *As* was successfully generated. An enzyme-linked immunosorbent assay was used to detect *As* by employing 96-well plates coated with scFv-conjugated affinity silk. *As* could be captured efficiently by glass wool coated with affinity silk in the column. Furthermore, the air-lift water filter equipped with the affinity silk-coated wool could considerably reduce the concentration of *As* in water and was estimated to have sufficient ability to trap a lethal dose of *As*. These findings show that the “affinity silk filter” is a potential device for the prophylaxis of aquatic animal diseases.

## Introduction

Infectious diseases are one of the most crucial problems in the aquaculture industry that cause massive economic losses. They are considered a major threat to the growth of the ornamental aquaculture industry^1,2^. The ornamental fish industry, which is a market for pet fish owners, is growing worldwide. Koi carp (*Cyprinus carpio*) is a popular ornamental and highly valuable fish. The most expensive koi carp was worth more than $1.8 million^3^. Thus, an effective method to prevent infectious diseases in this industry is essential. Many pathogens, including viruses, bacteria, and parasites, which infect ornamental fish, such as koi carp, goldfish, and marine or freshwater tropical fish, have already been identified. Hence, it is important to establish a disease control system in the owner’s private aquarium^2,4–6^. The main technologies for the diagnosis and prophylaxis are polymerase chain reaction (PCR) and vaccines, respectively, and they contribute to a large extent to the growth of the aquaculture industry^7^. Alternatively, immunological techniques to detect pathogens using either a monoclonal antibody (MAb) or a polyclonal antibody (PAb) also provide a useful means for detecting aquatic pathogens^8,9^. Antibodies against many aquatic pathogens have already been manufactured and used in diagnostic studies. However, the development of devices using antibodies for practical use is hampered by the associated high costs. Because diagnosis using PCR requires specialized equipment, such as a thermal cycler, PCR diagnostics is not feasible for individual pet owners.

Affinity reagents, such as traditional MAb and PAb and recombinant antibodies from animals and non-animals, are essential in biological and chemical research and medical applications. A novel affinity reagent termed “affinity silk” was developed using transgenic silkworm technology^10–12^^.^ Affinity silk is a silk protein containing the fibroin L-chain (FibL) fused to the single-chain variable fragment (scFv) that exhibits antigen specificity equivalent to conventional MAb. In addition, because the silk solution can be dialyzed, concentrated, freeze-dried, and processed into powder, the affinity silk can provide different types of devices for the detection of pathogens, such as ELISA plates and medical diagnostic kits. The costs of manufacturing devices that employ affinity silk are also substantially lower than those using traditional antibodies. Therefore, it is expected to be a potentially useful alternative to conventional MAbs.

Atypical *Aeromonas salmonicida* (*As*) is a pathogen that causes ulcer disease, one of the most severe diseases that affect the koi carp^13,14^. This disease causes progressive erosion of the skin and subsequent exposure of the underlying muscle, resulting in devastating disfigurement of the koi carp. The ulcer does not completely heal even if the illness subsides, and it thus causes a considerable reduction in the commercial value of koi carp. Because the administration of antibiotics can generate antibiotic resistance^15^, it is not an effective way of controlling ulcer disease. Therefore, for controlling ulcer disease, implementing “prophylaxis” rather than therapy is essential. In our previous study, *As*-specific polyclonal immunoglobulin (Ig)Y from chicken^16^ and *As*-specific mouse MAb were successfully produced^17^. Immersion of fish into aquarium water containing IgY prevented ulcer disease of koi carp, and immunofluorescence staining using MAb can identify *As* in fish having ulcer disease. *As*-specific MAb is variable in terms of the detection of *As* from ulcer disease^17^. Antibodies are reportedly useful tools for the prophylaxis and diagnosis of aquatic disease. The development of *As*-specific affinity silk is expected to result in the development of more inexpensive and versatile prevention and/or diagnostic devices for aquatic animals. In this study, *As*-specific affinity silk was produced and its novel usage, wherein it efficiently trapped pathogenic bacteria in water, was demonstrated.

## Results

### Expression of scFvs in mouse T-cells and *A. salmonicida* binding assay

**Figure 1:**
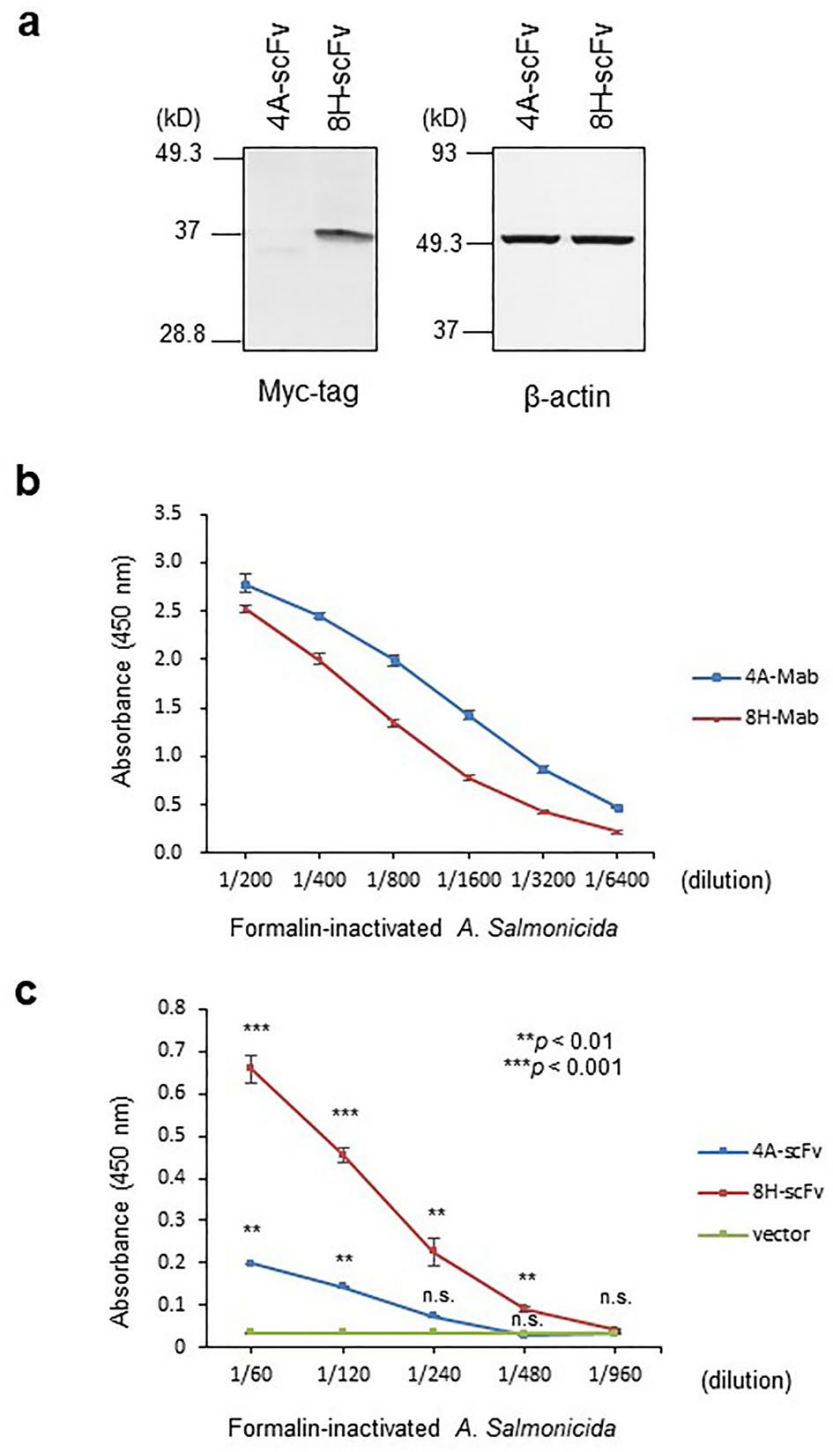
Anti-*As* scFvs expression in DO-11.10 cells and *As* binding assay. (**a**) Western blotting of anti-*As* 4A and 8H scFvs in transfected DO-11.10 T-cells. The immunoblots were probed with an anti-Myc-tag polyclonal antibody or anti-β-actin MAb. Full-length gels/blots are indicated in Supplementary Figure S2. (**b**) Binding assay of parental anti-*As* MAbs (4A and 8H) by ELISA. (c) Binding assay of anti-*As* scFvs by ELISA. DO-11.10 T-cell lysates transfected with DNA encoding anti-*As* 4A and 8H scFvs were used. The indicated dilutions of formalin-inactivated *A. salmonicida* were added to each well. Values are shown as mean ± standard error of duplicate reactions and are representative of three independent experiments. Asterisks represent a significant difference between the control (vector) and scFv (**p < 0.01; ***p < 0.001).

To generate scFv from hybridomas producing MAb against *As*, clones 4A and 8B^17^, a four-step PCR with appropriate primers for the amplification and assembly of the V_H_ and V_L_ regions was performed (Figure S1 and Table S1). We constructed two anti-*As* 4A and 8H scFvs, which were fused with the Myc-tag at the C-terminus. DO-11.10 murine T-cells were transiently transfected with these constructs. Western blot analysis detected adequate expression of 8H-scFv, although the expression of 4H-scFv with anti-Myc-tag antibody was extremely low. Equal amount of protein was loaded in each lane, as confirmed using Western blotting with an anti-β-actin antibody (Fig. 1a).

Both parental MAbs (4A and 8H) showed a strong binding activity to *As* (Fig. 1b). To assess the binding activity of anti-*As* scFvs to the target *As*, lysates from scFv gene-transfected T-cells were coated on 96-well plates and analyzed using ELISA. Anti-*As*-8H-scFv-coated wells displayed a strong reaction in an antigen concentration-dependent manner, whereas anti-*As*-4A-scFv-coated wells exhibited weaker ligand binding (Fig. 1c). The lower binding activity of the 4A-scFv is expected to result from the lower expression level and/or its instability in gene-transfected mouse T-cells. The 8H-scFv construct may be stable and capable of maintaining a suitable conformation for antigen binding when expressed in mammalian cells. Therefore, the 8H-scFv construct was used for generating transgenic silkworms.

### Transgenic silkworms produce scFv-conjugated affinity silk protein

**Figure 2:**
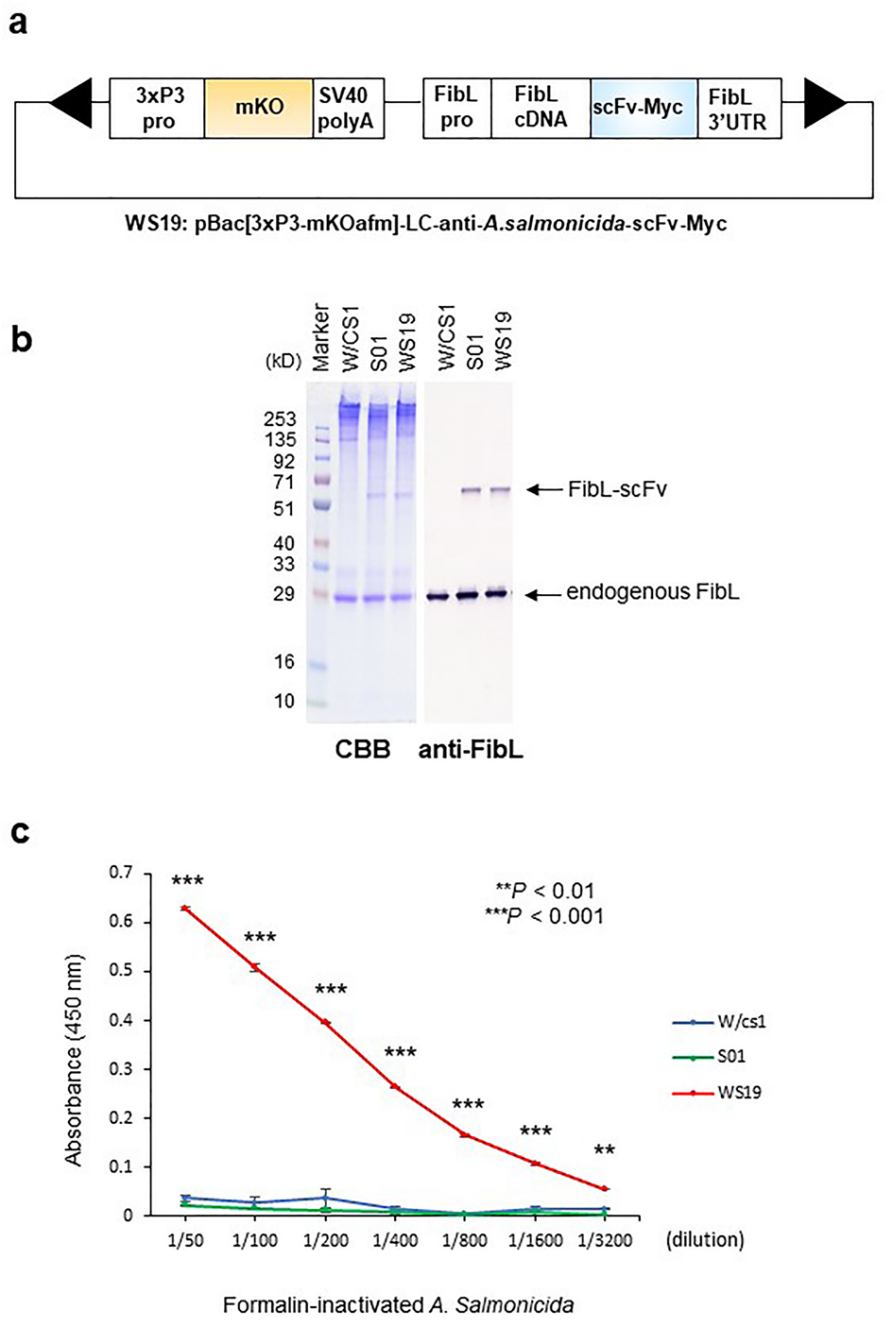
Construction of plasmid and production of affinity silk protein from transgenic silkworms. (**a**) Schematic representation of the DNA plasmids for WS19 transgenic silkworm strain. The plasmid contains expression units for the selection marker and recombinant protein between the *piggyBac* repeated terminal sequences (arrowheads). The 3xP3 promoter (3xP3pro), monomeric Kusabira-Orange gene (mKO), SV40 polyA signal sequence, fibroin L-chain promoter (FibLpro), cDNA of fibroin L-chain (FibL cDNA), the cDNA of anti-*As*-scFv fused with a Myc-tag sequence (scFv-Myc), and fibroin L-chain 3′-untranslated region (FibL-3’UTR) are shown. (**b**) SDS-PAGE and Western blotting of the expression of transgenes FibL-anti-*As*-scFv-Myc (WS19) and control FibL-anti-WASP-scFv-Myc (S01) in the silk solution. Silk solutions derived from wild-type (W/cs1), S01, and WS19 strains were separated by SDS-PAGE and stained with Coomassie brilliant blue. Immunoblots were probed with an anti-FibL polyclonal antibody. The molecular weights of endogenous FibL and FibL-scFvs were calculated at about 27 kD and 57 kD, respectively. (**c**) Specific binding was quantified by ELISA using 96-well plates coated with silk solution obtained from W/cs1, S01, or WS19 strains. The indicated dilutions of formalin-inactivated *A. salmonicida* were added to each silk solution-coated well. Values are shown as mean ± standard error of duplicate reactions and are representative of three indicated experiments. Asterisks indicate a significant difference between the control (W/cs1) and affinity silk (**p < 0.01; ***p < 0.001).

The DNA plasmid, pBac[3xP3-mKO]-LC-anti-*As*-scFv-Myc, for transgenic silkworm was constructed. The marker gene expression of the orange fluorescent protein (mKO) was under the control of the eye and nervous tissue-specific promoter 3xP3, and the expression of the anti-*As*-8H-scFv-Myc fused to the C-terminus of FibL was under the control of the FibL promoter. These expression units were incorporated into a *piggy*Bac transposon-derived vector^18^ (Fig. 2a). A transgenic silkworm strain, WS19, which spun silk containing FibL fused to the anti-*As*-8H-scFv, was generated. Wild-type W/cs1 and transgenic strain S01, which manufactures FibL-anti-WASP-scFv, were used as controls. The anti-Wiskott–Aldrich syndrome protein (WASP)-scFv construct originated from a MAb against WASP, which acts as an adaptor molecule in mammalian immune cells^12^. Expression of transgenes composed of the WS19 construct or control S01 construct was verified in each silk solution using sodium dodecyl sulfate–polyacrylamide gel electrophoresis (SDS-PAGE), followed by Coomassie brilliant blue staining and immunoblot analysis using an anti-FibL antibody (Fig. 2b). The scFv-conjugated FibL expression levels were estimated to be approximately 10% of those of endogenous FibL by densitometric analysis, which is comparable to the average expression levels noted in previous transgenic silk experiments. These results indicated that the anti-*As*-scFv construct fused with FibL is expressed to sufficient extent in silk fibers in the transgenic cocoon shells.

ELISA examined the specific binding of scFv-conjugated affinity silk solution for the target *As*. The absorbance of WS19 silk solution-coated wells increased as the concentration of *As* increased (Fig. 2c). As a negative control, the W/cs1 and S01 silk solution-coated wells did not show a positive reaction (Fig. 2c). These findings suggest that the anti-*As*-scFv construct fused to FibL in WS19 transgenic silkworm preserves the appropriate folding for antigen binding during dissolution, coating, and washing steps.

### Effect of affinity silk filter on capturing *A. salmonicida*

**Figure 3:**
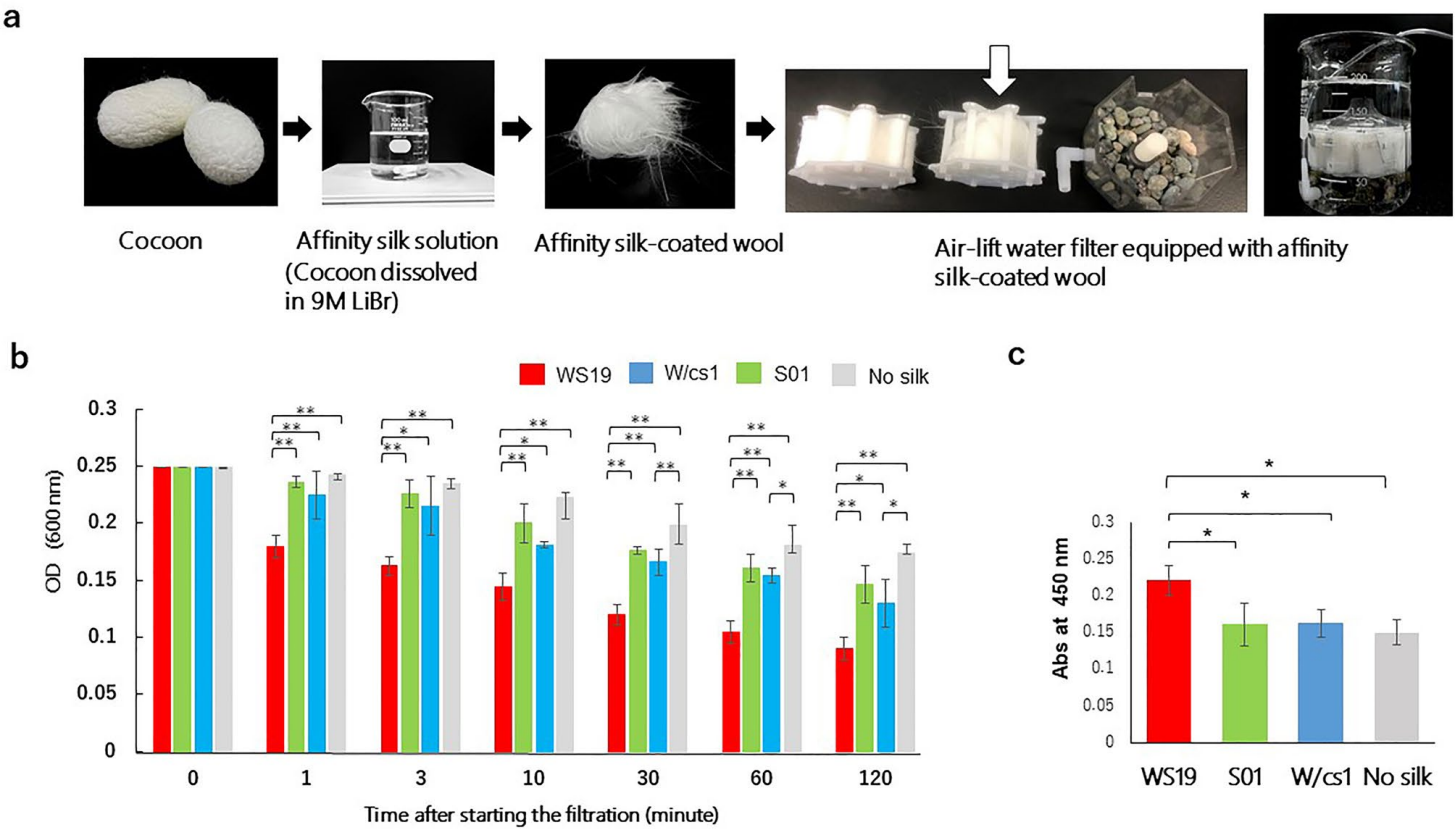
Efficacy of air-lift water filter equipped with affinity silk-coated wool on capturing *As*. (**a**) A schematic procedure for preparation of devices for capturing the bacteria. The affinity silk solution was prepared by dissolving the cocoons in 9 M Libr. It was then coated on the glass wool at a concentration of 0.2 mg/mL in 1 mM Tris–HCl (pH 8.0). Air-lift water filter and column were equipped with this affinity silk-coated glass wool. The white arrow indicates affinity silk-coated glass wool. WS19, W/cs1, S01, and No silk indicates anti-*As*-affinity silk, the wild type control, affinity silk control, and the control without any silk, respectively. (**b**) Efficacy of the filter units equipped with the affinity silk-coated glass wool on capturing *As*. The absorbance of bacterial suspension is shown at the indicated times during the filtration. Data are indicated as the means of the absorbance of the suspension (*N* = 3). Error bars indicate SD. Asterisks on each bar indicate significant differences (*p < 0.05, **p < 0.01). (**c**) Detection of *As* on the glass wool in the filter unit by ELISA. Data are shown as the means of the absorbance of the suspension (*N* = 3). Error bars indicate SD. Asterisks on each bar indicate significant differences (*p* < 0.05).

To detect the binding activity of affinity silk-coated glass wool to live *As*, a filtration assay was performed with water containing live bacteria (Fig. 3). As indicated in Fig. 3b, the filter with WS19 silk-coated glass wool (WS19 filter) could reduce *As* 1 min after filtration initiation, and the concentration of *As* in water continuously decreased within 120 min. The optical density of the suspension filtered using the WS19 filter was significantly lower than that of others at all sampling points. The filter with Ws/c1-coated glass wool (Ws/c1 filter) showed a significant reduction of *As* in water at 30, 60, and 120 min after filtration initiation. However, the Ws/c1 filter did not exhibit a significant decrease of *As* in a short time (within 10 min), whereas the WS19 filter could reduce it. These findings indicate that the WS19 filter can capture *As* as efficiently as other filters and the silk protein on the glass wool binds to the bacteria in a nonspecific manner.

To estimate *As* bound to the filters, each glass wool was removed and washed to remove the unbound bacteria. Next, the *As* captured on each wool was measured by ELISA. This was done by extracting the *As* bound to the glass wool. The amount of *As* trapped on the WS19-coated glass wool was significantly higher than that on S01 and W/cs1-coated glass wool and non-coated glass wool, showing that the WS19 filter absorbed *As* on the surface of the glass wool (Fig. 3c). No significant difference was noted between the absorbance of *As* on Ws/c1 and S01-coated glass wool, as well as that of *As* on the non-coated glass wool, which indicated that *As* on the wool was removed through the washing process. These results showed that the binding affinity of WS19-coated glass wool was higher than that of S01 and W/cs1-coated wool. To estimate the quantity of captured bacteria, the concentration of *As* was calculated using a standard curve of bacterial turbidity. WS19-coated wool filter could reduce the *As* count from 4.9 × 10^7^ colony forming units (CFU)/mL to 3.5 × 10^7^ CFU/mL in 1 min, indicating that one gram of WS19 coated wool can capture 1.96 × 10^9^ CFU of *As* per 1 min.

## Discussion

Because the aquatic environment is a medium ideal for the growth of microorganisms compared with air, the concentration of microbacteria in the water is much higher than that in air^19^. Therefore, aquatic animals are at a consistently higher risk of infection than terrestrial animals. Infectious diseases are a severe problem in the aquaculture industry world. Because antibodies are soluble proteins that act on the body fluid, affinity silk should bind to antigens in water efficiently. Therefore, affinity silk can effectively trap pathogens that live in environments containing water and its utility in preventing aquatic bacterial diseases was demonstrated.

Affinity silk is a “ready-made” reagent manufactured by transgenic silkworm technology that requires only a few processing steps such as dissolving the cocoons and dialyzing and drying the silk solution in assay plates^10–12^. Even though affinity silk is used in human medical and health industry^10–12^, no one has attempted to use it for the health of industrial animals. The filtration assay showed that not only the WS19 filter but also the wild-type (W/cs1) filters efficiently trapped bacteria compared with the filter that utilized the non-coated wool. However, no significant difference was noted between the bacteria absorbed on W/cs1,S01 glass wool, and non-coated glass wool, in the damping filter (Fig. 3c). This result suggested that the washing process removed the bacteria trapped on W/cs1 and S01 glass wool, and its binding was thus nonspecific and feeble. Alternatively, because the bacteria on WS19 glass wools were bound by their affinity toward antibodies, they were not released from the filter again. Thus, the affinity silk filter could rapidly and effectively clean live bacteria in water. We believe that it could be a powerful tool to prevent infectious diseases in aquatic animals.

Many infectious diseases have been found in ornamental fish species, including tropical fish and colored koi carp ^20,21^. Because individual pet owners cannot easily use vaccines in most countries because of technical and legal problems, administration of vaccines is not feasible for the owners. The administration of antibiotics generating resistant strains is a matter of concern, and using them in aquaculture is thus undesirable^15^. Furthermore, most infectious diseases cause the disfigurement of ornamental fish and a reduction in their worth, even if they are cured. Therefore, a filtration system to eliminate pathogens in water is expected to be established before fish are infected. Because the filter formation can be modified in a flexible manner, it is possible to process affinity silk filters for external upper box systems. Prospectively, if affinity silk against several infectious pathogens in a wide range of ornamental fish can be developed, a versatile disease prevention filter that is common to different ornamental fish may be developed. As affinity silk is a biological material, it would eventually be degraded in water. Thus, for it to be practically applied, further studies would need to investigate its degradation time and attempt to extend its use for the development of antiseptic treatment.

## Materials and methods

### Preparation of *A. salmonicida*

Atypical *A. salmonicida* (*As*) (strain B10F21, T1031) was cultured on heart infusion agar (HI; Nissui Pharmaceutical Co., Ltd., Tokyo, Japan) plates for 72 h at 20 °C. The colonies were identified as *As* using flow cytometry (FCM) using anti-*A. salmonicida* (anti*-As*) monoclonal antibody (MAb)^17^. The selected colonies were sub-cultured in 500-ml HI broth for 72 h at 20 °C. The concentration of bacteria was estimated by measuring the absorbance at 600 nm or CFU. Formalin-inactivation of the bacteria was conducted as described previously. Live and formalin-killed bacteria were used as antigens in the following experiments.

### Cloning and construction of anti-*A. salmonicida* scFvs

The hybridoma clones producing MAbs against *As*, 4A, and 8H, were established as previously described^17^. Total RNA from hybridoma cells was reverse transcribed using the SMART™ RACE cDNA amplification kit (Clontech, Mountain View, CA). PCR amplified cDNA fragments for the VH and VL regions with appropriate primers, and then mixed and assembled into the single-chain variable fragment (scFv)^22,23^ by four-step PCR amplification using appropriate primers containing linker sequence (Supplementary Figure S1 and Table S1). The resulting fragments were digested with *Not*I/*Xba*I and cloned into the pCAGGS-MCS expression vector^24,25^. The Myc-tag (EQKLISEEDL) was inserted into the *Xba*I*/Eco*RI site of all pCAG/anti-*As* scFv constructs. The result was that all anti-*As*-scFvs were fused with the Myc-tag at the C-terminus. The GenBank/EMBL/DDBJ accession numbers for the sequences of the cDNAs encoding V_H_ and V_L_ are 4A-V_H_, LC225752; 4A-V_L_, LC225755; 8H-V_H_, LC225753; 8H-V_L_, LC225756.

### Cells and electroporation

The murine T-cell hybridoma DO-11.10^26^ and hybridoma cells producing anti-*As* antibodies were maintained in RPMI1640 medium supplemented with 100 U/mL penicillin, 100 μg/mL streptomycin, 4 mM _L_-glutamine, 10-mM HEPES (all from Life Technologies, Carlsbad, CA, USA), and 10% fetal calf serum. DO-11.10 cells adjusted to a concentration of 5 × 10^6^ cells/400-μL culture medium with 1.25% dimethyl sulfoxide per cuvette were electroporated using a Gene Pulser (Bio-Rad, Hercules, CA, USA) with 20 μg plasmid DNA at 290-V and 960-μF.

### Construction of plasmids for transgenic silkworms

The cDNA fragment for anti-*As*-scFv-Myc was generated by PCR from pCAG/anti-*As*-8H-scFv-Myc using the sense primer #13 and reverse primer #14 (Supplementary Table S1). The PCR product was digested with *Bam*HI-*Sal*I, inserted between the FibL promoter region through the FibL coding region (FibLpro-FibL) and FibL 3’-untranslated region (FibL-3’-UTR), and the fused DNA fragment of FibLpro-FibL-anti-*As* -scFv-FibL-3’-UTR cloned into the *Asc*I-*Fse*I site of pBac[3XP3-mKOafm] vector, which was replaced DsRed2 DNA sequence in the original vector, pBac[3XP3-DsRed2afm]^27^, with monomeric Kusabira-Orange (mKO)^28^ as a selection marker. This construct was designated pBac[3XP3-mKOafm]-LC-anti- *As*-scFv-Myc.

### Generation of transgenic silkworm

Transgenic silkworms were generated as described elsewhere^2^ with minor modifications. The transgene plasmid and a helper plasmid vector, pHA3PIG, coding for *piggyBac* transposase^18^ were mixed at a concentration of 0.2 μg/μL each in 5 mM KCl and 0.5 mM phosphate buffer (pH 7.0) and injected into the fertilized eggs of the W/cs1 silkworm 5–10 h post-oviposition. The hatched larvae (G0) were reared on an artificial diet (Nihon Nosan, Kanagawa, Japan) at 25 °C until they developed into moths and were permitted to mate. Using fluorescent microscopy (MZ16FA, Leica Microsystems, Wetzlar, Germany), G1 embryos were screened for transgenic individuals with mKO expression 6–7 d after oviposition. Transgenic silkworms were reared and sib-mated for at least three generations. The experimental strain WS19 carried the transgene coding for the FibL fused with anti-*As*-8H-scFv-Myc. Control strain S01 carried the transgene coding for the FibL fused with anti-WASP scFv-Myc^10^.

### Solubilization of silk cocoons and preparation of silk solution

Three hundred milligrams of cocoon shells (2–3 shells) were chopped into 2–3-mm squares, washed in 5 mL 70% ethanol, and then dissolved in 3 mL 9 M LiBr and 90 mM Tris–HCl (pH 9.0). The cocoon squares were suspended for four hours at 37 °C until the silk proteins had completely dissolved. The solubilized silk solutions were adjusted to a 10 mg/mL concentration in 2 M LiBr as a stock solution.

### Immunoblotting

The gene-transfected DO-11.10 cells and silk solutions from W/cs1, S01, and WS19 strains were treated with 2 × SDS sample buffer, separated using 12% SDS-PAGE, and transferred to polyvinylidene difluoride membranes (Bio-Rad). The blots were blocked with Blocking One (Nacalai Tesque, Kyoto, Japan) for one hour at room temperature and incubated with anti-Myc polyclonal antibody (MBL, code no. 562, Nagoya, Japan), anti-β-actin rabbit MAb (Cell Signaling Technology, code no. #4970, Danvers, MA, USA), and anti-FibL polyclonal antibody (raised against a synthetic peptide representing FibL residues 67–80), followed by alkaline phosphatase-conjugated anti-rabbit immunoglobulins (Igs) (Dako, code no. D0306, Glostrup, Denmark). Immunoreactive proteins were detected using BCIP-NBT Solution Kit for Alkaline Phosphatase Stain (Nacalai Tesque).

### ELISA

The gene-transfected DO-11.10 cells were lysed using RIPA buffer (50 mM Tris–HCL pH 7.6, 150 mM NaCl, 1% Nonidet P-40, 0.5% sodium deoxycholate, and protease inhibitor cocktail; Nacalai Tesque) on ice for one hour. Cell lysates were centrifuged at 10,000 × *g* for ten minutes at 4 °C and the supernatants were used for ELISA. The stock silk solutions (10 mg/mL in 2 M LiBr) were diluted with 1 mM Tris–HCl (pH 8.0) to a concentration of 0.2 mg/mL. One hundred microliters of the cell lysate, culture supernatants from hybridoma cells, and diluted silk solutions were applied to 96 well plates and incubated overnight at 4 °C. After washing thrice with PBS, each well was blocked using ELISA Diluent (BioLegend, San Diego, CA, USA) at room temperature for one hour. After five washes with PBS and Tween 20, formalin-inactivated *As* (1.8 × 10^8^ CFU/mL) was diluted, applied to the wells, and incubated at room temperature for 90 min. Binding was detected using sequential incubation of plates with anti-*As*-4A MAb and HRP-conjugated anti-mouse IgG Fc (abcam, ab97265), followed by incubation with ELISA POD Substrate TMB solution (Nacalai Tesque). After color development, the reaction was stopped with 2N H_2_SO_4,_ and the absorbance was read at 450 nm using a microplate reader (iMark™ Microplate Reader; Bio-Rad).

### Filtration assay using glass wool coated with affinity silk

One gram of glass wool (Masuda Corporation, Osaka, Japan) was soaked in a solution with or without 4 ml 0.2 mg/mL WS19, W/cs1, S01 in 1 mM Tris–HCl (pH 8.0), and was incubated at 4℃ overnight. After incubation, the glass wool was taken out and was washed with pure sterile water. This glass wool was then packed inside the commercial air-lift water filter (Suisaku eight-core mini; Suisaku Co., Ltd. Japan) (Fig. 3a). 4.9 × 10^7^/mL CFU of live *As* was suspended in 1.68 L distilled water and was equally divided into 12 beakers (140 mL/beaker). The filter units were inserted into each beaker, and the filtered bacterial solutions were collected at 0, 1, 3, 10, 30 min, 1, and 2 h after starting aeration. The turbidity of the obtained samples was measured using a spectrophotometer at 600 nm. Images of the filtered water were also taken at 0, 10, 30 min, 1, and 4 h of filtration. It was confirmed that the affinity silk was absorbed on the wool by Western blotting using anti-Myc-tag-pAb as the primary antibody and anti-IgG (H + L-chain) (Rabbit) pAb-HRP as secondary (MBL life science) (data not shown).

The glass wool in the filtration units was ejected, thoroughly washed with 100 mL of distilled water in a beaker, and was treated with 2 mL 0.01% Triton X 100-PBS for ten minutes. The elute was obtained after centrifugation, diluted ten times in carbonate-bicarbonate buffer, and was subjected to an indirect ELISA. Anti-*As* MAb 5H (1:5000 dilution of mouse ascites) and anti-IgG Fc Fragment antibody-HRP (MBL life science, Japan) of 1:50,000 dilution was used as the primary and secondary antibodies, respectively. Other steps in this ELISA were followed as described above. These experiments were repeated thrice independently.

### Statistical analysis

The Student’s t-test analyzed sample pairs. Multiple samples were evaluated using one-way ANOVA with Tukey’s test. Differences were considered significant when *p* < 0.05.

## Supplementary Information

Supplementary Information.

## Data Availability

The sequences of the cDNAs encoding V_H_ and V_L_ in the present study is available at Nucleotide database of CBI (https://www.ncbi.nlm.nih.gov/nuccore/, Accession Number: LC225752; LC225755: LC225753: LC225756).
