# Supplementary material for: Development of a filter device for the prevention of aquatic bacterial disease using a single-chain variable fragment (scFv)-conjugated affinity silk
Source: Sci Rep. 2022 Jun 8;12:9475. doi: 10.1038/s41598-022-13408-6 (PMC9177605; doi:10.1038/s41598-022-13408-6)
Supplement: Supplementary file 1 — Supplementary Information. [file 41598_2022_13408_MOESM1_ESM.docx]

Supplementary Information Figure S1

　　　　
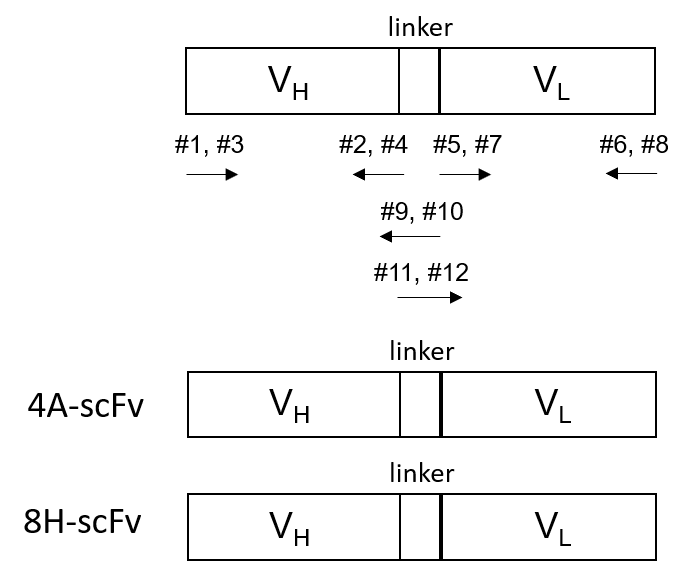


Figure S1. Construction of anti-*A. salmonicida*-scFvs by cloning the variable region of immunoglobulin heavy and light chains from hybridoma cells producing anti-*A. salmonicida* monoclonal antibody. The arrows represent the primers used to amplify the sequences encoding antibody fragments.

The isotype of 4A and 8H MAb was identified as IgG2b and κ using a mouse MAb isotyping kit, IsoStrip (Roche Diagnostics, Mannheim, Germany). We performed a four-step PCR to generate appropriate cDNA fragments encoding the V_H_ and V_L_ regions. Total RNA from hybridoma cells was reverse-transcribed using the SMART^TM^ RACE cDNA Amplification Kit (Clontech, Palo Alto, CA, USA). The cDNA fragments for the V_H_ and V_L_ regions were generated by PCR using isotype-specific primers (heavy chain, IgG2b: sense primer 5’-AAGCAGTGGTATCAACGCAGAGTACGCG-3’ and reverse primer 5’GGACAGGGGTTGATTGTTGAAATGGG-3’; light chain, κ: sense primer 5’- AAGCAGTGGTATCAACGCAGAGTACGCG-3’ and reverse primer 5’-CCTGTTGAAGCTCTTGACAATGGGTG-3’). The second PCR amplification was performed with the following primer sets: 4A-V_H_, sense primer #1 and reverse primer #2; 8H-V_H_, sense primer #3 and reverse primer #4; 4A-V_L_, sense primer #5 and reverse primer #6; 8H-V_L_, sense primer #7 and reverse primer #8. The third PCR products were amplified using the following primer sets: 4A-V_H_-linker, sense primer #1 and reverse primer #9; 8H-V_H_-linker, sense primer #3 and reverse primer #10; linker-4A-V_L_, sense primer #11 and reverse primer #6; linker-8H-V_L_, sense primer #12 and reverse primer #8. The third PCR products were mixed in the following combinations: 4A-V_H_-linker and linker-4A-V_L_, 8H-V_H_-linker and linker-8H-V_L_ and then the fourth PCR amplification was performed with the following primer sets: 4A-scFv, sense primer #1 and reverse primer #6; 8H-scFv, sense primer #3 and reverse primer #8. The fourth PCR products were digested with *Not*I-*Xba*I and cloned into the pCAGGS-MCS expression vector.

Table S1.

Specific oligonucleotide primers used to amplify anti-*A. salmonicida* 4A and 8H scFv

#1: 5’-CGAATgcggccgcGCCACCATGCAGGCTTATCTACAGCAGTCTGGG-3’

#2: 5’-CAGAACCACCACCCCCTGCAGAGACAGTGACCAG-3’

#3: 5’-CGAATgcggccgcGCCACCATGGAGGTCCAGCTGCAGCAGTCTGGA-3’

#4: 5’-CAGAACCACCACCCCCTGAGGAGACTGTGAGAGTG-3’

#5: 5’-GGTGGAGGAGGTTCTCAAATTGTTCTCACCCAGTCTC-3’

#6: 5’--3’CTAGtctagaCCGTTTCAGCTCCAGCTTGGTCC-3’

#7: 5’-GGTGGAGGAGGTTCTGACATCCAGATGACTCAGTCTC-3’

#8: 5’-CTAGtctagaCCGTTTGATTTCCAGCTTGGTGCC-3’

#9: 5’-GAACAATTTGAGAACCTCCTCCACCTGATCCTCCACCTCCAGAACCACCACCCCC-3’

#10: 5’-TCTGGATGTCAGAACCTCCTCCACCTGATCCTCCACCTCCAGAACCACCACCCCC-3’

#11: 5’-TGTCTCTGCAGGGGGTGGTGGTTCTGGAGGTGGAGGATCAGGTGGAGGAGGTTCT-3’

#12: 5’-AGTCTCCTCAGGGGGTGGTGGTTCTGGAGGTGGAGGATCAGGTGGAGGAGGTTCT-3’

#13: 5’-CTCCggatccGGCACCATGGAGGTCCAGCTGCAG-3’

#14: 5’- CCGgtcgacCTACAGGTCCTCCTCGCTGATCAG-3’

Lowercase letters indicate restriction site for *Not*I (gcggccgc), *Xba*I (tctaga), *Bam*HI (ggatcc), and *Sal*I (gtcgac).

Supplementary information Figure S2. (Full-length gels/blots)


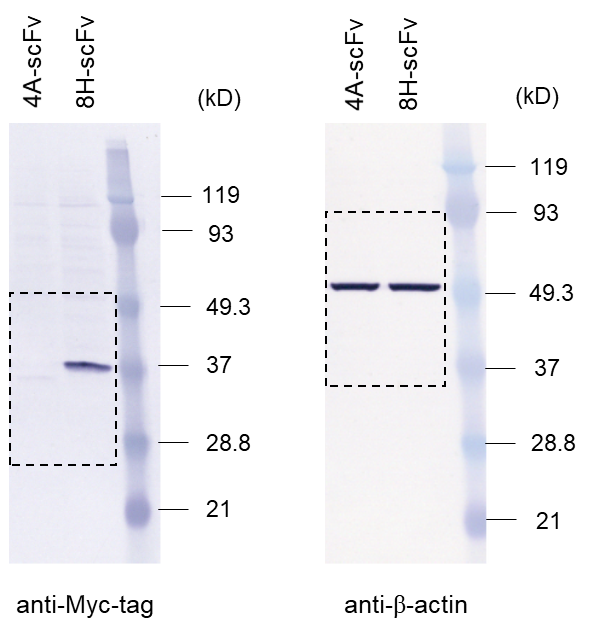


Figure S2. Westernblot of anti-*A. salmonicida* 4A and 8H scFvs in transfected DO-11.10 cells. The immunoblots were probed with anti-Myc-tag polyclonal antibody or anti-β-actin MAb. Each dotted line area indicates cropped blots in the main figures.
